# Impact of pulsed electromagnetic fields on intervertebral disc degeneration: a systematic review of preclinical and clinical evidence

**DOI:** 10.3389/fragi.2026.1840672

**Published:** 2026-05-19

**Authors:** Francesca Veronesi, Francesca Salamanna, Lucia Martini, Deyanira Contartese, Riccardo Ghermandi, Laura Marchese, Alessandro Gasbarrini, Gianluca Giavaresi

**Affiliations:** 1 Surgical Sciences and Technologies, IRCCS Istituto Ortopedico Rizzoli, Bologna, Italy; 2 Spine Surgery Unit, IRCCS Istituto Ortopedico Rizzoli, Bologna, Italy; 3 Department of Biomedical and Neuromotor Sciences, Alma Mater Studiorum University of Bologna, Bologna, Italy

**Keywords:** aging, inflammation, intervertebral disc degeneration, low back pain, pulsed electromagnetic fields, systematic review

## Abstract

**Introduction:**

Low back pain (LBP) is a leading cause of disability worldwide, with intervertebral disc degeneration (IDD) as a major contributor. IDD is strongly associated with aging and is characterized by cellular senescence, extracellular matrix degradation, and chronic low-grade inflammation, all of which impair disc homeostasis. Current treatments often provide limited or transient relief, prompting interest in non-invasive, biologically active approaches such as pulsed electromagnetic fields (PEMFs). This systematic review evaluates the evidence on PEMF therapy for IDD, including *in vitro*, *in vivo*, and clinical studies, and summarizes mechanisms of action, efficacy, and limitations.

**Methods:**

A systematic search of PubMed, Scopus, and Web of Science (2000–2025) was conducted following PRISMA guidelines. Eligible studies included cell, animal, or clinical models of IDD treated with PEMFs versus controls, reporting significant outcomes. Risk of bias was assessed using QUIN, SYRCLE, and RoB 2.0 tools.

**Results:**

Sixteen studies were included: 7 in vitro, 4 in vivo, 4 clinical RCTs, and 1 combined study. *In vitro*, PEMFs reduced pro-inflammatory cytokines, MMPs, and apoptosis, while promoting ECM synthesis, BMP signaling, and autophagy/SIRT1 pathways, mechanisms implicated in age-related degeneration. *In vivo*, PEMFs improved disc structure, reduced inflammation, and enhanced functional recovery, with timing influencing outcomes. Clinically, PEMFs were safe and showed potential benefits in pain, function, and mobility, especially alongside physiotherapy, although results were inconsistent and influenced by age-related variability. Methodological limitations were common.

**Discussion:**

PEMF therapy appears safe and biologically active, with potential to modulate key processes of IDD and aging, including inflammation, senescence, and impaired autophagy. However, current evidence remains insufficient for clinical recommendations. Standardized protocols, mechanistic studies, and long-term trials stratified by age are needed.

**Systematic Review Registration:**

https://www.crd.york.ac.uk/PROSPERO/view/CRD420251247528.

## Introduction

Low back pain (LBP) is a leading cause of disability worldwide and represents a major socioeconomic burden. Up to 80% of individuals experience LBP during their lifetime, and a significant proportion develop chronic or recurrent symptoms (Yang et al., 2025; GBD, 2021 Low Back Pain Collaborators, 2023).

Intervertebral disc degeneration (IDD) is considered the main pathological substrate of LBP (Zabek et al., 2025; Sun et al., 2022). The intervertebral disc (IVD) consists of the nucleus pulposus (NP), annulus fibrosus (AF), and cartilage endplate (CEP), which together ensure load distribution and spinal mobility (Zabek et al., 2025; Sun et al., 2022).

IDD is a multifactorial and progressive condition in which aging represents a central and unavoidable driver, interacting with genetic predisposition, mechanical loading, lifestyle, and environmental factors (Karchevskaya et al., 2023). At the molecular level, degeneration is characterized by sustained inflammation, extracellular matrix (ECM) breakdown, cell senescence and apoptosis, and neurovascular ingrowth, all contributing to pain generation (Yang et al., 2024). Pro-inflammatory cytokines such as interleukin1β (IL1β), IL6, and tumor necrosis factor α (TNFα) activate catabolic enzymes, including metalloproteinases (MMPs) and ADAMTS, leading to proteoglycan loss, collagen disruption, and disc dehydration (Costachescu et al., 2022; Ren et al., 2025; Shnayder et al., 2023; Empere et al., 2023).

Current management strategies for disc-related disorders remain suboptimal. Conservative treatments often provide only temporary symptom relief, while surgical options are invasive, costly, and associated with variable long-term outcomes (Zhou et al., 2024). These limitations have stimulated interest in non-invasive therapies targeting the biological mechanisms of degeneration (Dorsi et al., 2024; Ikwuegbuenyi et al., 2025).

Pulsed electromagnetic fields (PEMFs) are a non-invasive biophysical therapy with documented anti-inflammatory, analgesic, and tissue-modulating effects in several musculoskeletal disorders (Kaadan et al., 2025; Hackel et al., 2025). PEMFs have been shown to influence cell proliferation, differentiation, matrix synthesis, and cytokine release (Zou et al., 2017; Song et al., 2024; Liu et al., 2024; Li et al., 2025). However, their role in IDD, particularly regarding neuroinflammation and disc regeneration, is not fully defined (Caliogna et al., 2022; Wang et al., 2024). In addition, PEMF parameters and mechanisms of action remain poorly standardized. At the molecular level, PEMFs have been shown to inhibit pro-inflammatory signaling cascades, particularly the nuclear factor kappa B (NF-κB) pathway, leading to reduced expression of cytokines such as IL1β, IL6, and TNFα, which are key mediators of intervertebral disc degeneration. In parallel, PEMFs can decrease oxidative stress by reducing reactive oxygen species (ROS) production and enhancing cellular antioxidant defenses (Tang et al., 2019).

In addition to their anti-inflammatory effects, PEMFs promote anabolic processes by stimulating extracellular matrix (ECM) synthesis, including aggrecan and type II collagen (COLL II), which are essential for disc structural integrity. These effects appear to be mediated, at least in part, by the activation of bone morphogenetic protein (BMP) signaling pathways. Furthermore, recent evidence suggests that PEMFs may regulate autophagy and cellular senescence through pathways involving SIRT1, thereby contributing to cell survival and maintenance of disc homeostasis (Zheng et al., 2022).

Overall, these mechanisms support the potential role of PEMFs as a biologically active, non-invasive approach capable of modulating key processes underlying intervertebral disc degeneration.

This systematic review synthesizes current *in vitro*, *in vivo*, and clinical evidence on PEMF therapy for IVD-related disorders, evaluates proposed biological mechanisms, and highlights limitations and future research directions.

## Methods

This systematic review was designed using the PICOS framework (Population, Intervention, Comparison, Outcomes, Study design). Specifically, studies were considered if they met the following criteria: cells, animal model or patients with IVD degeneration (Population) who underwent PEMF stimulation (Intervention), compared with untreated controls (Comparison), reporting pathogenic mechanisms (Outcomes), in *in vitro, in vivo* or clinical studies (Study Design).

A systematic search was conducted in November 2025 across three databases (PubMed®, Scopus, and Web of Science™), following the Preferred Reporting Items for Systematic Reviews and Meta-Analyses (PRISMA) guidelines (Page et al., 2021). The search terms used were: (“pulsed electromagnetic fields”[MeSH Terms] OR “pulsed electromagnetic field therapy” OR “PEMF” OR “electromagnetic stimulation”) AND (“intervertebral disc”[MeSH Terms] OR “intervertebral disk” OR “intervertebral disc degeneration” OR “disc degeneration” OR “nucleus pulposus” OR “annulus fibrosus”).

Filters were applied to limit results to studies published in English between 2000 and 2025. The reference lists of the selected articles and pertinent reviews were manually examined as well, in order to identify any additional eligible studies. Duplicate records were removed using EndNote®, and titles and abstracts were screened independently by two authors (FV and FS). Studies that did not meet the inclusion criteria were excluded. Any disagreements were resolved through discussion, or, if necessary, with the involvement of a third reviewer (GG). Full-text articles of the remaining studies were assessed for eligibility, and reference lists were also examined for additional relevant studies.

Data extraction was performed independently by FV and FS using a standardized extraction form, and the collected information is summarized in Tables 1–3. The protocol was registered in PROSPERO (cod. CRD420251247528).

**TABLE 1:** *In vitro* studies.

References	Cell source	Experimental conditions	PEMF parameters	Methods	Main findings
Guarnaccia et al. (2025)	Human NP cells (degenerative IVD, n = 10)	Control; IL1β; PEMF; IL1β+PEMF	75 Hz, 2.0 ± 0.5 mT, 4 h/day × 5 days	MTT, RT-PCR, WB, ROS, NO, GSH	IL1β: ↑ ROS, NO, IL6, MMPs; ↓ ECM. PEMF: ↓ oxidative stress and inflammation; ↑ ACAN, COLL II, PPARG
Miller et al. (2016)	Human NP and AF cells (degenerative IVD)	Control ± IL1α ± PEMF	15 Hz burst (3,850 Hz carrier), 4 h/day × 7 days	Live/Dead, microarray	IL1α: ↑ cytokines, MMPs, NF-κB; ↓ ECM. PEMF: ↓ IL1α-induced IL17A, MMP2; no cytotoxicity
Tang et al. (2019)	Bovine AF cells	Control; IL1α; PEMF; IL1α+PEMF	15 Hz burst, 4 h/day	WB, IHC	IL1α: ↑ p65 and p38 PEMF: ↓ NF-κB activation and nuclear translocation
Okada et al. (2013a)	Human AF cells (degenerative IVD)	Control; PEMF; BMP2; BMP2+PEMF	15 Hz, 8 h/day x 3 days	RT-PCR, WB, GAG assay	PEMF + BMP2 synergistically: ↑ ECM (ACAN, COLL II) and BMP signaling
Okada et al. (2013b)	Human AF cells (degenerative IVD)	Control; PEMF; Noggin; Noggin + PEMF	15 Hz, 8 h/day x 3 days	RT-PCR, WB, GAG assay	PEMF: ↑ ECM and BMP signaling; effects abolished by BMP inhibitor (Noggin)
Zheng et al. (2022a)	Human NP cells (degenerative IVD, n = 8)	Control; PEMF; PEMF+3-MA; PEMF + EX-527	15 Hz, 4 h/day × 4 days	CCK-8, RT-PCR, WB, IF, TEM	PEMF: ↑ SIRT1, autophagy, ECM; ↓ MMP3. Effects reversed by autophagy/SIRT1 inhibition
Lee et al. (2010)	Human NP cells (degenerative IVD)	Control; PEMF	30 Hz, 1.8 mT, 72 h	MTT, RT-PCR, DNA synthesis	PEMF: ↑ DNA synthesis and proliferation; no cytotoxicity
Zou et al. (2017)	Rat NP cells (healthy)	Control; PEMF (0.5–3 A/m)	2 Hz, 30 min twice/day x 7 days	CCK-8, WB, ELISA	PEMF: ↓ IL1β, TNFα in a dose-dependent manner; no cytotoxicity

**TABLE 2:** *In vivo* studies.

References	Animal model	Experimental groups	PEMF parameters	Methods	Main results
Chan et al. (2019)	72 female SD rats; tail disc puncture (Co6–Co9)	Sham; no treatment; PEMF	3.846 kHz, 4 h/day × 7 days	Histology, RT-PCR, ELISA	No treatment: ↑ IL1β, IL6, TNFα; PEMF restored levels toward control
Chan et al. (2021)	19 female SD rats; L4–L5 puncture	Sham; no treatment; PEMF	3.846 kHz, 3 h/day × 7 weeks	Treadmill analysis	PEMF: ↑ gait recovery; ↓ motor impairment
Zheng et al. (2022a)	15 rats; tail puncture (Co6–Co8)	Sham; no treatment; PEMF	15 Hz, 4 h/day × 2 months	MRI, histology	PEMF: ↓ Pfirrmann score; improved disc structure
Kermani et al. (2014)	24 male Wistar rats; tail puncture	Sham; no treatment; PEMF (10 Hz); PEMF (100 Hz)	10–100 Hz, 2 h/day × 3 months	Immunoblot	PEMF: ↓ caspase-3 and Bax/Bcl2 ratio (anti-apoptotic effect)
Zheng et al. (2022b)	20 male SD rats; tail puncture (Co6–Co10)	Sham; no treatment; PEMF daytime; PEMF nighttime	15 Hz, 4 h/day × 2 months	MRI, microCT, histology, WB	PEMF: ↑ disc height, ECM (COLL II, ACAN); ↓ MMP3; daytime more effective

**TABLE 3:** Clinical studies.

References	Study design	Population	Experimental groups	PEMF parameters	Outcomes	Main results
Elshiwi et al. (2019)	RCT	50 pz chronic non-specific LBP	Physiotherapy; Physiotherapy + PEMF	50 Hz, 20 G, 20 min/session × 1 month	VAS, ODI, ROM, AEs	PEMF: ↓ pain and disability; ↑ ROM; no AEs
Hattapoglu et al. (2019)	RCT	64 pz, cervical disc herniation	Physiotherapy; Physiotherapy + PEMF	50 Hz, 0.6 mT, 20 min/day × 3 weeks	3 months after treatment: VAS, NPDS	Both improved; PEMF group showed greater pain reduction
Foley et al. (2008)	RCT	323 pz undergoing ACDF	Control; PEMF	4 h/day × 3 months	12 months after treatment: Fusion rate, VAS, NDI, SF-12, AEs	No significant differences between groups
Omar et al. (2012)	RCT	40 pz, lumbar radiculopathy	Control; PEMF	0.07–4 kHz, 20 min/day × 3 weeks	VAS, ODI, SSEP	PEMF: ↓ pain and disability; improved SSEP parameters

Abbreviations: Ref., references; NP, nucleus pulposus; IVD, intervertebral disc; IL, interleukin; PEMF, pulsed electromagnetic field; WB, Western blot; NO, nitric oxide; AF, annulus fibrosus; ECM, extracellular matrix; ACAN, aggrecan; COLL II, collagen type II; MMPs, matrix metalloproteinases; ROS, reactive oxygen species; GSH, glutathione; PPARG, peroxisome proliferator-activated receptor gamma; IHC, immunohistochemistry; BMP, bone morphogenetic protein; GAG, Glycosaminoglycans; 3-MA, 3-methyladenine; EX-527, Selisistat; SIRT1, silent information regulator 1; IF, immunofluorescence; TEM, transmission electron microscope; MRI, magnetic resonance imaging; TNFα, tumor necrosis factor α; BAX, Bcl-2-associated X protein; Bcl2, B-cell lymphoma 2; LBP, low back pain; ACDF, anterior cervical discectomy and fusion; VAS, visual analogue scale; ODI, oswestry disability index; ROM, range of motion; AEs, adverse events; NPDS, neck pain and disability scale; NDI, neck disability index; SSEP, somatosensory evoked potentials.

### Risk of bias assessment

The risk of bias of the included *in vitro* studies was evaluated using the QUIN Tool, a checklist designed for the critical appraisal of laboratory-based experimental research (Sheth et al., 2024). The tool assesses 12 key domains, including clarity of study objectives, sample size and selection, presence of appropriate control groups, methodological transparency, operator details, randomization, outcome measurement methods, blinding, statistical analysis, and completeness of reporting. Each criterion was scored on a scale of 0–2 (0 = not satisfied, 1 = partially satisfied, 2 = adequately satisfied), with the option to mark items as not applicable (NA) when a specific criterion did not pertain to a study. Total scores were converted into percentages to classify the overall risk of bias for each study: >70% indicated low risk, 50%–70% moderate risk, and <50% high risk.

The risk of bias of the included animal studies was assessed using SYRCLE’s Risk of Bias tool (Hooijmans et al., 2014), which is adapted from the Cochrane framework to address methodological features specific to preclinical research. The tool assesses risk of bias in several areas, such as sequence generation, baseline comparability, allocation concealment, random housing, blinding of researchers and outcome evaluators, incomplete outcome data, selective outcome reporting, and other possible bias sources. For each area, studies were classified as having low, high, or unclear risk of bias, according to the details provided in the reports.

As regards clinical randomized clinical trial (RCT) studies, the Cochrane Risk of Bias 2.0 (RoB 2) tool was applied, which evaluates five domains: (1) bias arising from the randomization process, (2) bias due to deviations from intended interventions, (3) bias due to missing outcome data, (4) bias in measurement of the outcome, and (5) bias in selection of the reported result. Each domain was rated as low, moderate, or high risk of bias, following the Cochrane Handbook recommendations (Sterne et al., 2019).

All assessments of all the tools were performed independently by two reviewers (FV and FS), with discrepancies resolved through discussion or consultation with a third reviewer (GG).

## Results

### Included and excluded studies

**FIGURE 1:**
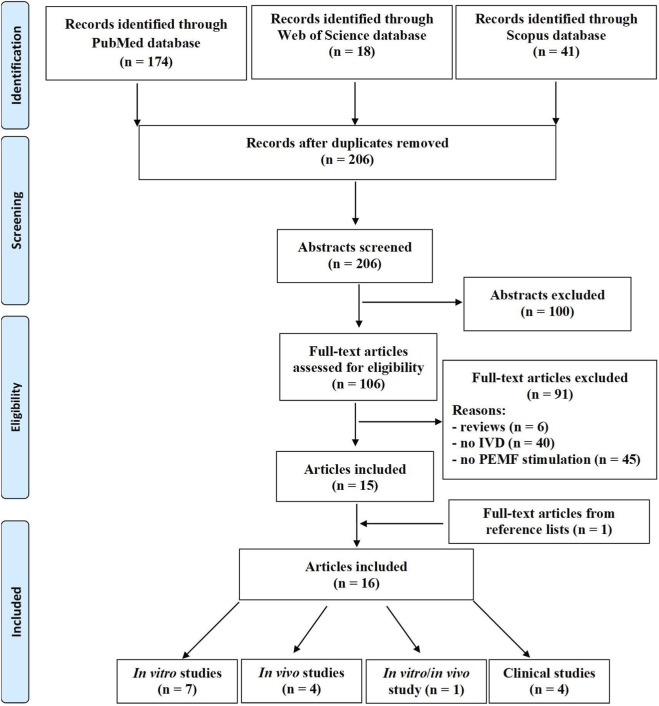
The PRISMA flow diagram for the systematic review detailing the database searches, the number of abstracts screened, and the full texts retrieved.

The initial database search identified a total of 233 articles, 174 from PubMed, 18 from Web of Science, and 41 from Scopus. After removing 27 duplicates, 206 studies were screened by title and abstract (Figure 1). Of these, 100 were excluded for being not inherent to the topic. The remaining 106 were assessed for their eligibility and 91 were further excluded for various reasons: 6 were reviews articles, 40 did not focus on IVD, and 45 did not employ PEMF stimulation. Ultimately, 15 studies met the inclusion criteria, and 1 additional study was identified from reference screening, resulting in a total of 16 studies included in this systematic review.

Of these, 7 were *in vitro* studies (Guarnaccia et al., 2025; Miller et al., 2016; Tang et al., 2019; Okada et al., 2013a; Okada et al., 2013b; Lee et al., 2010; Zou et al., 2017), 4 were animal *in vivo* studies (Chan et al., 2019; Chan et al., 2021; Kermani et al., 2014; Zheng et al., 2022a), 4 were clinical ones (Elshiwi et al., 2019; Hattapoglu et al., 2019; Foley et al., 2008; Omar et al., 2012), and 1 were both *in vitro* and *in vivo* study (Zheng et al., 2022b).

**FIGURE 2:**
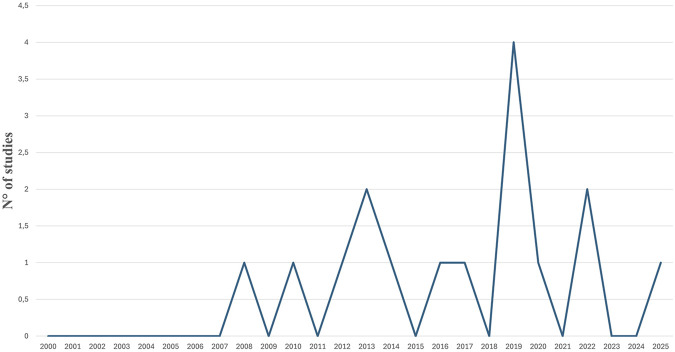
Number (N) of articles per year.

In the last 25 years of literature, the 16 included studies were published between 2008 and 2025, with a peak in 2019 (n = 4 studies) and no study in 2009, 2011, 2015, 2018, 2021, 2023, and 2024 (Figure 2).

### *In vitro* studies

**FIGURE 3:**
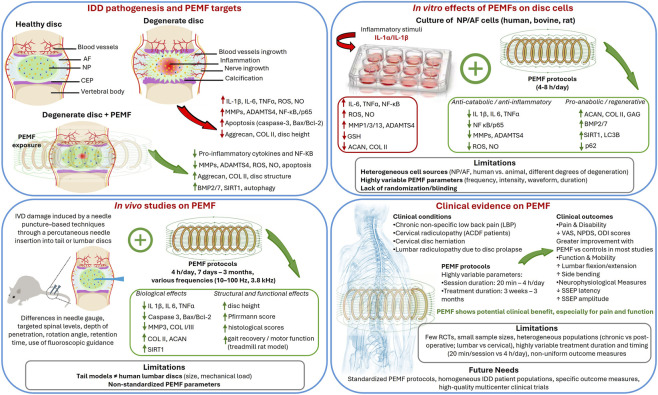
Graphical summary of the effects of PEMFs on intervertebral disc degeneration across experimental and clinical studies.

Among the *in vitro* studies (Guarnaccia et al., 2025; Miller et al., 2016; Tang et al., 2019; Okada et al., 2013a; Okada et al., 2013b; Lee et al., 2010; Zou et al., 2017; Zheng et al., 2022b), n = 4 used NP cells from human donors with degenerative IVD (D-IVD), originating from: degenerative lumbar spondylolisthesis (DLS) (Guarnaccia et al., 2025), Pfirrmann grade ≤2 (Miller et al., 2016), Pfirrmann grade 3 or 4 (Lee et al., 2010), and lumbar disc herniation (Zou et al., 2017). One study used NP cells from healthy rats (Zheng et al., 2022b). AF cells were obtained from human D-IVD samples, with Pfirrmann grade ≤2 (Miller et al., 2016), from human disc with unspecified pathology (Okada et al., 2013a; Okada et al., 2013b), or from bovine tissue (Tang et al., 2019). The Pfirrmann grading system is an MRI-based classification of intervertebral disc degeneration ranging from Grade I (normal morphology) to Grade V (severe degeneration), based on disc structure, signal intensity, distinction between nucleus and annulus, and disc height. This scale provides a standardized and reproducible method for evaluating degenerative changes in lumbar discs (Pfirrmann et al., 2001). PEMF stimulation parameters and exposure durations varied across studies (Table 1; Figure 3), with the most common protocol involving 4 or 8 h of stimulation per day over periods of 3–7 days (Guarnaccia et al., 2025; Miller et al., 2016; Tang et al., 2019; Okada et al., 2013a; Okada et al., 2013b; Zheng et al., 2022b).

In three studies, cells were pre-treated with IL1β (Guarnaccia et al., 2025) or IL1α (Miller et al., 2016; Tang et al., 2019), to induce an inflammatory microenvironment, prior to PEMF exposure. Interleukin treatment significantly upregulated inflammatory mediators, including cytokines, MMPs, enzymes, apoptosis-related markers, growth factors, pain markers, ROS, nitric oxide (NO), p65 and p38, while significantly reducing antioxidant protein, endogenous anti-inflammatory factors, and ECM proteins (Guarnaccia et al., 2025; Miller et al., 2016; Tang et al., 2019). PEMF stimulation counteracted these effects, reducing IL1α-induced IL17A and MMP2 gene expression in NP cells, nuclear factor kappa-light-chain-enhancer of activated B cells (NF-κB) activity in AF cells, and p65 nuclear translocation, without compromising cell viability.

In another three studies, cells were pre-treated with bone morphogenetic protein 2 (BMP2) (Okada et al., 2013b), Noggin (a BMP inhibitor) (Okada et al., 2013a), or with autophagy inhibitor 3-methyladenine (3-MA) and SIRT1 inhibitor EX-527 (Zheng et al., 2022b), before PEMF stimulation. Both BMP2 and PEMF significantly increased BMP2 and BMP7 gene and protein expression, with the combination producing ever greater effects also with increased ECM protein production compared with BMP2 alone (Okada et al., 2013b). Differently, PEMF effects were completely abolished in the presence of Noggin (Okada et al., 2013a). PEMFs significantly upregulated SIRT1 expression and autophagy; however, 3-MA reversed the PEMF-mediated enhancement of ECM production, and EX-527 inhibited PEMF-induced autophagy as well as ECM synthesis (Zheng et al., 2022b).

Finally, two studies applied PEMF without any cells pre-treatment (Lee et al., 2010; Zou et al., 2017). PEMF significantly enhanced DNA synthesis without reducing cell viability or causing cytotoxicity (Lee et al., 2010) and decreased IL1β and TNFα levels in an intensity-dependent manner (0.5–3 A/m) (Zou et al., 2017).

### *In vivo* studies

A total of 150 rats were included across the five studies (91 females, 44 males, and 15 with unspecified sex). Among them, 58 rats received PEMF stimulation, while 92 served as non-stimulated controls or sham animals (Table 2; Figure 3). IVD damage was induced using needle puncture–based techniques in all studies, although the specific procedures varied. While all models involved percutaneous needle insertion into tail or lumbar discs, they differed in needle gauge, targeted spinal levels, depth of penetration, rotation angle, retention time, use of fluoroscopic guidance, and in some cases the addition of further procedures such as NP removal or air injection.

As observed in the *in vitro* studies, PEMF parameters also differed considerably across the *in vivo* experiments. Daily exposure ranged from 2 to 4 h, with overall treatment durations spanning from 7 weeks to 3 months.

In three studies, PEMF stimulation significantly reduced inflammatory mediators, including IL1β, IL6 and TNFα, restoring their levels to those of healthy rats, when compared with injured untreated animals (Chan et al., 2021). These improvements were accompanied by a faster recovery of motor function, normalization of gait speed, and reduced gait disturbances relative to non-treated rats (Chan et al., 2021). Furthermore, PEMF significantly improved both histological and radiological features of the IVD (Zheng et al., 2022b).

In two studies, PEMF was applied either at different frequencies (10 and 100 Hz) (Kermani et al., 2014) and at different times of the day, daytime (7:00–11:00 a.m.) and nighttime (19:00–23:00) (Zheng et al., 2022a). With both frequency groups, PEMF significantly decreased caspase-3 and Bax/Bcl2 levels compared with non-stimulated controls (Kermani et al., 2014). Additionally, PEMF increased disc height, enhanced ECM protein expression, and reduced MMP levels in both daytime and nighttime stimulation, with daytime exposure producing significantly greater effects than nighttime one (Zheng et al., 2022a).

### Clinical studies

All four clinical studies included were RCTs (Elshiwi et al., 2019; Hattapoglu et al., 2019; Foley et al., 2008; Omar et al., 2012) (Table 3; Figure 3). A total of 477 patients were enrolled: 240 received PEMF therapy, 57 underwent conventional physical therapy, and 180 received no treatment. No patients were lost to follow-up. The overall male-to-female ratio was 0.92 (229 males/248 females), with a mean patient age of 40.7 years. The clinical conditions represented across the studies included chronic non-specific LBP (Elshiwi et al., 2019), compressed cervical nerve root with symptomatic radiculopathy in patients undergoing anterior cervical discectomy and fusion (ACDF) (Hattapoglu et al., 2019), cervical disc herniation (Foley et al., 2008), and lumbar radiculopathy secondary to disc prolapse (Omar et al., 2012).

Consistent with findings from the *in vitro* and *in vivo* literature, PEMF parameters varied considerably across trials. Exposure times ranged from 20 min per session (Elshiwi et al., 2019; Foley et al., 2008; Omar et al., 2012) to 4 h per day (Hattapoglu et al., 2019), while overall treatment durations spanned from 3 weeks to 3 months.

In the first two studies, conventional physiotherapy was provided with or without PEMF stimulation (Elshiwi et al., 2019; Hattapoglu et al., 2019). Conventional treatment consisted of transcutaneous electrical nerve stimulation (TENS) (100 Hz) and ultrasound (1 MHz, continuous mode, 1.5 W/cm^2^) applied several times per week, combined with a one-month exercise program targeting strengthening and stretching the abdominal, back, pelvic, and lower-limb muscles in three weekly sessions (Elshiwi et al., 2019) or TENS combined with hot pack (HP) therapy. No adverse events were reported, and both conventional therapies associated or not with PEMF led to significant reductions in Visual Analog Scale (VAS) and Neck Pain and Disability Scale (NPDS) scores over time. However, PEMF produced significantly greater improvements in pain, disability, right and left lumbar side bending, and significantly increased lumbar flexion and extension (Elshiwi et al., 2019), as well as a significantly greater reduction in VAS compared with conventional therapy alone (Hattapoglu et al., 2019).

In the remaining two studies, PEMF was evaluated without comparison to other physical therapies (Foley et al., 2008; Omar et al., 2012). In patients undergoing ACDF, PEMFs did not significantly improve fusion rate, adverse events (AEs), VAS, Neck Disability Index (NDI), and Short Form-12 (SF-12) outcomes compared with ACDF alone at final follow-up (Foley et al., 2008). Conversely, PEMF significantly reduced VAS and modified Oswestry Disability Index (OSW) scores, decreased left Somatosensory Evoked Potentials (SSEP) latency, and increased right SSEP latency and amplitude, as well as left SSEP amplitude in comparison with untreated patients (Omar et al., 2012).

### Risk of bias assessment

**FIGURE 4:**
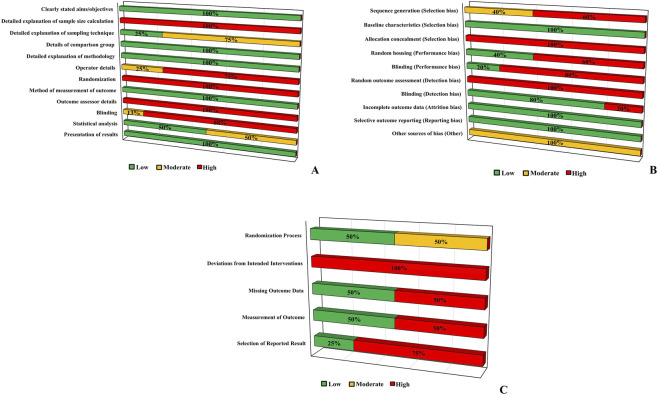
Results of risk of bias assessment for *in vitro* studies (QUIN Tool) **(A)**, *in vivo* studies (SYRCLE’s Risk of Bias) **(B)**, and clinical studies (Cochrane Risk of Bias 2.0) **(C)**.

The *in vitro* studies exhibited generally robust methodological reporting, although notable gaps persisted (Figure 4A). Several domains received uniformly low-risk ratings, including the clarity of aims and objectives, the description of comparison groups, the explanation of methodology, the measurement of outcomes, the details of outcome assessors, and the presentation of results, all rated as low risk in 100% of the studies. These findings suggest good transparency in fundamental study design and outcome measurement procedures. However, other domains highlighted persistent methodological weaknesses. The explanation of sampling technique was frequently incomplete, with 75% of studies assessed as moderate risk. Operator details and blinding were also critical weaknesses, receiving high-risk ratings of 75% and 88%, respectively, underscoring limited reporting or implementation of blinding procedures and potential operator-related variability. Randomization was similarly rated as high risk across all studies, reflecting the common lack of random allocation procedures in laboratory settings.

Overall, the risk of bias assessment revealed substantial methodological limitations across animal studies (Figure 4B). Most domains had a high risk of bias, particularly those related to selection, performance, and detection bias. Allocation concealment and random outcome assessment were consistently rated at high risk, indicating poor reporting or implementation of randomization procedures. Sequence generation showed only a small proportion of studies rated as low or moderate risk. Random housing, blinding in performance and detection rated as low or moderate risk. Domains such as baseline characteristics, incomplete outcome data, and selective reporting demonstrated a low-risk judgement, while other potential sources of bias also showed moderate risk. These findings highlight pervasive deficiencies in methodological rigor and reporting quality, which may affect the reliability and internal validity of the included preclinical evidence.

Clinical studies demonstrated substantial methodological limitations across several domains (Figure 4C). The domain “Deviations from Intended Interventions” was judged as high risk in 100% of the studies, reflecting consistent concerns regarding adherence to the assigned protocols. Similarly, “Missing Outcome Data,” “Measurement of Outcome,” and “Selection of the Reported Result” showed high-risk ratings in 50%–75% of the studies, indicating frequent issues related to incomplete datasets, potential detection bias, and selective reporting. Only the “Randomization Process” displayed a more favorable distribution, with 50% of the studies classified as low risk, although the remaining half still fell into moderate risk, suggesting inconsistencies in adequate sequence generation or allocation concealment. Overall, the clinical evidence base was characterized by recurrent high-risk judgments that may meaningfully influence internal validity.

## Discussion

**FIGURE 5:**
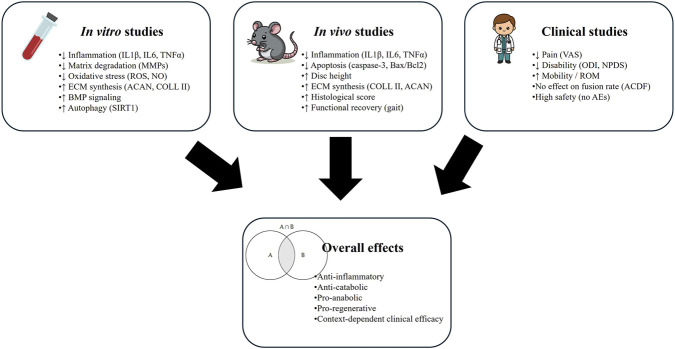
Schematic representation of the current evidence on the effects of PEMFs on IDD, integrating *in vitro*, *in vivo*, and human studies.

This systematic review synthesized and critically evaluated the current evidence on the effects of PEMFs on IDD, integrating *in vitro*, *in vivo*, and human studies. Collectively, the findings indicate that PEMFs may modulate key pathogenic mechanisms underlying IDD, particularly inflammation, matrix degradation, apoptosis, and disc structural deterioration (Figure 5). Nonetheless, substantial variability across study designs, stimulation protocols, and outcome measures limits the ability to draw definitive conclusions and underscores the need for more rigorous and standardized research.

The included *in vitro* studies offer important mechanistic insights into how PEMFs may influence disc cell biology. A consistent finding across models is that PEMF exposure attenuates pro-inflammatory and catabolic pathways that are central to IDD progression. Studies using IL1α or IL1β pretreatment demonstrated that PEMFs significantly downregulated cytokines, MMPs, oxidative stress markers, and transcription factors such as NF-κB and p65 (Guarnaccia et al., 2025; Miller et al., 2016; Tang et al., 2019), which are known to drive the degenerative cascade within the NP and AF (Chen et al., 2024). These results suggest potential for PEMFs to interrupt the self-amplifying inflammatory cycle typical of degenerating discs (Xu et al., 2021).

Importantly, several studies also reported pro-anabolic effects of PEMFs. Enhanced synthesis of ECM components, including aggrecan and collagen, was observed across models (Guarnaccia et al., 2025; Miller et al., 2016; Okada et al., 2013a; Okada et al., 2013b; Zheng et al., 2022b), with some studies showing synergistic activity between PEMFs and BMP2/BMP7 pathways (Okada et al., 2013a; Okada et al., 2013b). For instance, the combination of PEMF and BMP2 stimulation resulted in greater increases in ECM protein production than BMP2 alone (Okada et al., 2013a), whereas blockade of BMP signaling via Noggin fully abolished PEMF-induced effects (Okada et al., 2013a). These findings point to a mechanistic link between PEMFs and BMP-mediated regenerative signaling, supporting the hypothesis that PEMFs may promote matrix restoration through modulation of developmental or reparative pathways.

Another relevant mechanistic axis implicated in the reviewed studies is autophagy and SIRT1 activity (Zheng et al., 2022b). One study demonstrated that PEMFs enhanced autophagic processes and SIRT1 expression, both of which have been associated with protection against cell senescence and degeneration in disc tissue (Zhang et al., 2025). Inhibition of autophagy or SIRT1 abolished PEMF-induced ECM synthesis, further supporting the idea that these molecular pathways contribute to PEMF-mediated disc regeneration (Zheng et al., 2022b).

Together, the *in vitro* evidence reveals a multifaceted mode of action, whereby PEMFs reduce inflammation and apoptosis while simultaneously promoting anabolic and cytoprotective responses.

Despite these promising findings, interpretation is complicated by the substantial heterogeneity in cell sources, pretreatment conditions, PEMF exposure times, and stimulation parameters. Notably, most studies used stimulation durations of 4–8 h per day (Guarnaccia et al., 2025; Miller et al., 2016; Tang et al., 2019; Okada et al., 2013a; Okada et al., 2013b; Zheng et al., 2022a), but frequencies, intensities, and waveforms differed considerably and were often not directly comparable. Additionally, only a subset of studies used human disc cells (Guarnaccia et al., 2025; Miller et al., 2016; Okada et al., 2013a; Okada et al., 2013b; Lee et al., 2010; Zheng et al., 2022b), varying degrees of degeneration and different underlying pathologies. The lack of standardized models limits the ability to extrapolate these findings to clinical settings and highlights the need for harmonized experimental protocols.

Consistent with *in vitro* findings, the *in vivo* studies included in the systematic review demonstrated that PEMFs exert significant anti-inflammatory and regenerative effects following disc injury (Chan et al., 2019; Chan et al., 2021; Kermani et al., 2014; Zheng et al., 2022a; Zheng et al., 2022b). Across multiple models employing needle-puncture techniques, PEMF-treated animals exhibited reduced levels of IL1β, IL6, and TNFα (Chan et al., 2019), approaching levels observed in healthy untreated controls. These biochemical improvements were accompanied by enhanced disc height preservation (Zheng et al., 2022b), improved NP and AF histological architecture (Zheng et al., 2022a; Zheng et al., 2022b), and increased ECM protein expression (Zheng et al., 2022a) compared with injured but untreated animals. Such findings strongly support the notion that PEMFs modulate pathogenic inflammatory and degradative processes at the tissue level, and that these molecular changes translate into measurable structural benefits.

Functional outcomes further reinforced the biological significance of these changes. PEMF-treated animals displayed faster recovery of gait parameters and motor function compared with controls, suggesting that PEMFs may mitigate pain-related or neurologic deficits induced by disc injury (Chan et al., 2021). Although behavioral assessments remain indirect proxies for disc-related pain in rodents, these findings are consistent with the analgesic and neuroprotective effects reported for PEMFs in other musculoskeletal and inflammatory conditions (Hackel et al., 2025; Zhou et al., 2025).

Interestingly, two studies explored the effects of varying stimulation parameters, providing preliminary insights into dose- and time-dependency (Kermani et al., 2014; Zheng et al., 2022a). Different frequencies (10 Hz vs. 100 Hz) produced similar reductions in caspase-3 and Bax/Bcl-2 levels, suggesting that multiple PEMF settings may exert anti-apoptotic effects (Kermani et al., 2014). However, when stimulation was applied at different times, daytime versus nighttime, PEMF exposure produced significantly greater regenerative outcomes if administered during daytime (Zheng et al., 2022a). While the mechanisms underlying such chronobiological differences remain unknown, these findings underscore the potential importance of treatment timing and biological rhythms in modulating PEMF responsiveness.

Nevertheless, direct comparison across studies remains difficult due to methodological inconsistencies. Needle-puncture techniques varied in gauge size, penetration depth, rotation, retention time, and targeted disc levels. Stimulation durations ranged from 7 weeks (Chan et al., 2021) to 3 months (Kermani et al., 2014), and PEMF frequencies, intensities, and exposure patterns were seldom standardized. These differences highlight the need for systematic parameter optimization, ideally supported by mechanistic experiments and dose–response analyses. In addition, most *in vivo* studies used rats in which degeneration is induced in the tail discs rather than in lumbar discs (Chan et al., 2019; Kermani et al., 2014; Zheng et al., 2022a; Zheng et al., 2022b). This presents important limitations because small disc size, different anatomical and biomechanical loading conditions compared with the human lumbar spine reduce the translational relevance of such models (Wang et al., 2022).

The four clinical RCTs included in this systematic review provide preliminary but valuable evidence regarding the potential usefulness of PEMFs in treating disc-related pain and dysfunction (Elshiwi et al., 2019; Hattapoglu et al., 2019; Foley et al., 2008; Omar et al., 2012). Importantly, PEMF therapy was well tolerated in all studies, with no reported AEs, supporting its safety as a non-invasive adjunctive modality (Elshiwi et al., 2019; Foley et al., 2008).

Two studies evaluated PEMFs in combination with conventional physiotherapy, TENS, ultrasound, hot packs, and structured exercise, and found that adding PEMFs produced significantly greater improvements in pain relief, disability scores, and lumbar range of motion compared with physiotherapy alone (Elshiwi et al., 2019; Hattapoglu et al., 2019). These findings suggest possible therapeutic synergy between PEMFs and standard conservative treatments, potentially through enhanced anti-inflammatory and analgesic effects.

However, clinical outcomes were not uniformly positive across all patient populations. In patients undergoing ACDF for cervical radiculopathy, PEMF stimulation did not improve fusion rates or functional outcomes compared with surgery alone (Foley et al., 2008). This may reflect the fundamentally different biological context of acute postoperative healing compared with chronic inflammatory degeneration (Wang et al., 2022). Conversely, in a study of patients with lumbar radiculopathy due to disc prolapse, PEMF treatment yielded significant improvements not only in pain and disability scores but also in somatosensory evoked potentials, suggesting a potential neuromodulatory effect (Omar et al., 2012).

These contrasting findings highlight the importance of patient selection, timing of intervention, and underlying pathology in determining PEMF efficacy. Chronic degenerative conditions, characterized by sustained inflammation, may be more responsive to PEMF modulation than acute postoperative settings, dominated by wound healing and mechanical stabilization. Furthermore, differences in treatment duration, ranging from 20-min sessions (Elshiwi et al., 2019) to 4-h daily (Foley et al., 2008) exposure, complicate direct comparison and make it difficult to identify optimal therapeutic windows.

Another limitation of the current clinical evidence is the relatively small number of trials and the heterogeneity of outcome measures used. Pain scores, disability indices, fusion rates, neurophysiological tests, and patient-reported outcomes varied widely across studies. Standardized and validated measures specific to disc degeneration would strengthen the reliability of future findings.

Finally, it should be noted that cervical spine studies, particularly those involving fusion procedures, reflect a distinct therapeutic context compared to degenerative lumbar disc disease, as they primarily address postoperative bone healing rather than disc regeneration. Therefore, their findings should be interpreted with caution when extrapolating to degenerative disc conditions.

Taken together, the studies included in this systematic review provide preliminary evidence that PEMFs may modulate key molecular and structural processes associated with IDD and improve clinical outcomes in selected contexts. The consistent anti-inflammatory, anti-apoptotic, and anabolic effects observed across *in vitro*, *in vivo*, and clinical studies support a plausible rationale for their potential role in slowing degenerative changes. However, several important challenges remain. Marked heterogeneity in PEMF parameters precludes identification of optimal stimulation settings, and mechanistic pathways are still incompletely understood. Translation from preclinical to clinical settings is further limited by anatomical, biomechanical, and disease-related differences between animal models and humans, as well as by the limited number of high-quality clinical RCTs. In addition, methodological limitations were pervasive across study types. *In vitro* studies frequently lacked randomization and blinding, animal studies showed high risk of bias and poor adherence to reporting ARRIVE (Animal Research: Reporting of *In Vivo* Experiments) guidelines (Percie du Sert et al., 2020), and clinical trials were affected by protocol deviations, missing data, and outcome measurement bias. Collectively, these limitations underscore the need for more rigorous, standardized, and adequately powered studies to define the clinical value of PEMF therapy.

To conclude, PEMF therapy is a safe, non-invasive intervention with demonstrated biological activity in models of intervertebral disc degeneration. By modulating inflammation, apoptosis, and matrix metabolism, PEMFs may represent a potential disease-modifying approach rather than solely symptomatic treatment. However, current evidence is insufficient for definitive clinical recommendations. Future research should focus on parameter optimization, mechanistic clarification, chronobiological effects, and high-quality clinical trials to establish long-term efficacy and clinical relevance.

## Data Availability

The original contributions presented in the study are included in the article/supplementary material, further inquiries can be directed to the corresponding author.
